# Influence of CO_2_‑Regenerative Film Properties in Enhancing C_2+_ Products Selectivity While Mitigating CO_2_ Crossover

**DOI:** 10.1021/acs.energyfuels.6c01357

**Published:** 2026-06-29

**Authors:** Ashok Kumar Ummireddi, Ananya Vasudevan, Jithu Raj, Mohammad Fahim Yasir, Jingjie Wu

**Affiliations:** † Department of Chemical and Environmental Engineering, 2514 University of Cincinnati, Cincinnati, Ohio 45221, United States; ‡ Department of Chemical Engineering, Indian Institute of Technology Dharwad, Dharwad, Karnataka 580011, India

## Abstract

Zero-gap anion-exchange membrane electrode assembly (AEMEA) electrolyzers operating in alkaline media face challenges such as CO_2_ crossover and salting-out. The bipolar membrane electrode assembly (BPMEA) electrolyzer, using DI water as the electrolyte, addresses both CO_2_ crossover and salting-out issues. As cations, which are crucial in stabilizing the CO_2_R intermediates, are absent in the electrolyte, cations embedded in the AEM play an important role in dictating the CO_2_R selectivity and activity in BPMEA systems. So far, no systematic study has been conducted on the influence of cations embedded in AEMs on CO_2_ selectivity and activity in BPMEA systems. Moreover, BPMEA systems impose an additional challenge: low stability due to delamination of the bipolar membrane caused by CO_2_ regeneration at the membrane-membrane interface. To enhance the stability of the electrolyzer, a simple yet highly reproducible strategy for coating a porous CO_2_ regenerative film on smooth Nafion 117 is demonstrated in this work, along with a systematic study of four different commercially available AEMs, composed of different cations and cation densities, for use in combination with Nafion 117 and copper catalyst at the cathode. We found that the PiperION membrane delivers selectivity and activity comparable to those of alkali-metal cations, owing to the enhanced local electric field resulting from the combined effects of a high positive charge on the N atom of the piperidinium cation and the high ion-exchange capacity. Further, we studied the influence of the thickness of the PiperION porous layer over Nafion 117 on CO_2_R selectivity and found that 70 μm is the minimum thickness to achieve maximum C_2+_ products selectivity, reduced the CO_2_ crossover to 5% from 25% at 4 SCCM and 150 mA cm^–2^, and was stable for operation beyond 100 h. The selectivity of this system, compared with AEMEA, and stability outperformed both AEMEA and BPMEA. This study helps design more effective BPMs to inhibit CO_2_ crossover while enabling stable and selective electrochemical CO_2_ reduction to C_2+_ hydrocarbons.

## Graphical abstract

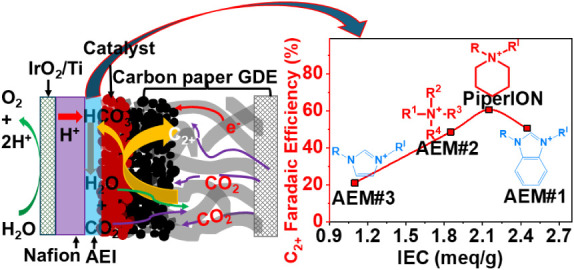

## 1 Introduction

The need for energy storage from renewable and intermittent energy resources, such as solar, and the increase in CO_2_ emissions demand the commercialization of electrochemical CO_2_ reduction (ECO_2_R) to chemicals/fuels.^1^ Among thermochemical, electrochemical, and photochemical CO_2_ conversion technologies, electrochemical CO_2_ conversion technology is a promising approach, as the cost of renewable electricity is cheap and continues to decrease.^2–4^ Zero-gap membrane electrode assemblies (MEAs) incorporating gas diffusion electrodes (GDEs) are commonly employed to overcome the solubility and diffusion limitations of CO_2_ in aqueous electrolytes, achieving industrially relevant current densities at lower full-cell voltages due to their low overall resistance.^5,6^ Anion-exchange membrane electrode assembly (AEMEA) electrolyzers are widely used to maintain an alkaline environment for enhancing ECO_2_R while suppressing the parasitic hydrogen evolution reaction (HER). OH^–^ ions generated during ECO_2_R chemically react with CO_2_ to form carbonate and bicarbonate anions, which are then transported from the cathode to the anode through the anion exchange membrane (AEM). If we consider ethylene to be the only product during ECO_2_R, only 25% of the CO_2_ undergoes electrochemical conversion, while 75% is theoretically wasted and crosses over the AEM due to the electromigration of carbonate and bicarbonate anions.^7^ Regeneration of CO_2_ from these ions is an energy-intensive process. In addition, AEMEA electrolyzers face other scale-up challenges, such as salt formation, which results from the reaction of alkaline cations (e.g., K^+^) that diffuse from the anode to the cathode with carbonate and bicarbonate ions. To mitigate CO_2_ crossover, acidic MEA systems are proposed and show promise in inhibiting it; however, the salting issue persists due to the use of high concentrations of alkali metal cations. Since cations are essential for stabilizing CO_2_R intermediates, a high concentration of K_2_SO_4_ is added to the acidic solution.^8^ This “salting-out” results in a buildup of gas pressure in the line, reduces CO_2_ flow through the channels, and hampers cell stability. Moreover, the use of alkaline electrolytes, such as KOH, results in water flooding in the GDE due to electro-osmotic drag and salting-out. As the solubility of CO_2_ in water is limited (33 mM at ambient conditions), water flooding in the cathodic compartment reduces CO_2_ flux to the catalyst and lowers the Faradaic efficiency (FE) for CO_2_-reduction products due to concurrent HER activity.

Recent studies suggest that the bipolar membrane (BPM) electrode assembly electrolyzer addresses the above-mentioned challenges. O’Brien et al. demonstrated single-pass CO_2_ conversion of 85% by coating a permeable CO_2_ regeneration layer using an anion exchange ionomer (AEI) (Aemion^+^ AP1-CNN5-00-X) on the electrocatalyst surface.^9^ Since the catalyst-loaded GDE has a rough surface, the possibility of crack formation during film formation is high, resulting in proton transport through the cracks and dominating HER activity. Xu et al. introduced a zero-gap BPM electrolyzer operating in forward bias mode with a modified integrated channel layer between the anion exchange layer (AEL) and cation exchange layer (CEL), called the microchanneled solid electrolyte (MSE), to inhibit CO_2_ crossover and improve stability by preventing salt precipitation. This design enabled 51% C_2_H_4_ selectivity at 100 mA cm^–2^, with only 3% CO_2_ crossover and no sign of degradation over 200 h of operation.^10^ The acidic integrated channel layer interface between the AEL and CEL was used for CO_2_ regeneration while maintaining a higher pH at the cathode microenvironment for ECO_2_R. The quaternary ammonium cation in the AEM assisted in stabilizing the ECO_2_R intermediates. The MSE is hindered by its complex manufacturing process and high costs. Brückner et al. demonstrated 80% CO selectivity at 100 mA cm^–2^ over a NiNC single-site catalyst by using Nafion/Sustainion (anode/cathode) BPM in a forward bias mode.^11^ The positively charged quaternary nitrogen groups in Sustainion allow for similar performance in the presence of metal cations. However, this AEM design lacks microporosity, which may lead to delamination between Nafion and Sustainion. Stability analysis was not reported in their study. Xie et al. prepared a PiperION/Nafion (anode/cathode) BPM electrolyzer under a reverse bias mode with a stationary buffer catholyte layer (65 μm thick 0.5 M K_2_SO_4_ layer) and achieved a current density of 200 mA cm^–2^ and 42% C_2_H_4_ FE at a CO_2_ flow rate of 1.42 SCCM cm^–2^. The cell’s stability was limited to 50 h, maybe due to potential flooding of the GDL and delamination at the BPM interface.^12^ Kim et al. designed a modified BPMEA cell using silver nanowires as cathode catalysts with a porous solid electrolyte (PSE) in place of the BPM. The PSE was formed by a permeable, ion-conducting sulfonated polymer electrolyte placed between the cathode and anode for ion exchange. This design incorporates an additional layer (i.e., PSE between AEM and CEM), which introduces an extra resistance and energy penalty to the electrolyzer operation.^13^ She et al. demonstrated a highly stable pure-water-fed BPMEA system by simply stacking AEM and PEM together in the MEA system under an elevated operating temperature (e.g., 60 °C). The elevated temperature vaporizes the accumulated water at the BPM interface, thereby increasing electrolyzer stability.^14^ However, elevated temperatures promote the thermal degradation of AEM, ultimately leading to the long-term instability of the electrolyzer. Furthermore, all these studies utilized commercial AEMs to produce BPM. These commercially available AEMs differ in their cation backbones, and their influence on product selectivity and activity has not been systematically studied to date. It is unclear which AEM should be used in combination with Nafion/PEM to configure the BPM. In addition, the above-mentioned studies face challenges such as reproducibility, scale-up, and stability (Table S1). To address these challenges, a simple yet high-reproducibility strategy for coating an AEI-based CO_2_ regenerative layer on a smooth Nafion 117 membrane is demonstrated in this work. Furthermore, this work systematically studies commercially available AEMs/AEIs for use in BPM, as the organic cations embedded in these materials play a crucial role in stabilizing the CO_2_^–^ anion or its derived intermediates and in dictating product selectivity.^15^ This study analyzes the behavior of four commercial AEMs in the BPM cell configuration with the CEM (Nafion 117) using a copper catalyst at the cathode in a forward bias configuration to curb CO_2_ feed loss by acidifying carbonate and bicarbonate ions in a stable electrolyzer. Among the tested AEMs, PiperION (AEM#4) delivered C_2+_ FE and performance comparable to alkali metal cations, likely due to the high local electric field generated by the membrane-embedded, nonconjugated piperidinium cations. The high local electric field generated by PiperION can be attributed to the combined effects of a high Bader/positive charge on the N atom of the piperidinium cation, which is a measure of the strength of the cation in stabilizing CO_2_R intermediates, and a high ion-exchange capacity, which is a measure of the cation density. Subsequently, PiperION AEI was employed to coat onto CEM to form a porous CO_2_ regenerative layer. A 70 μm-thick AEI porous layer (AEIPL) yielded the optimum results, i.e., a minimum HER and a lower cell voltage. Also, a 70 μm-thick AEIPL-based BPMEA cell reduced the CO_2_ crossover to 5% from 25% at 4 SCCM and 150 mA cm^–2^. The cell was stable for 100 h of operation. Overall, the selectivity of AEIPL-based BPMEA cells compared to AEMEA cells, as well as their stability, outperformed both AEMEA and conventional BPMEA cells.

## 2 Experimental Details

### 2.1 Chemicals and Materials

Iridium­(III) chloride hydrate (99.8%) was purchased from Alfa Aesar. Ethanol, 200 proof (100%), was purchased from Fisher Chemical. Platinized titanium felt, AEM#1, AEM#2, and Gas Diffusion Layer (GDL, Sigracet 39 BB) were purchased from the Fuel Cell Store. Oxalic acid (98%), deuterium oxide (99.9%), silver nanoparticles (25 nm), copper nanoparticles (25 nm), trisodium phosphate (96%), isopropyl alcohol (98%), potassium hydroxide (≥85%), potassium bicarbonate (99.7%), dimethyl sulfoxide (99.9%), and phenol (97%) were purchased from Sigma-Aldrich. PiperION (5 wt % solution) was purchased from Versogen. Nafion 117 was purchased from TheSix Tech. AEM#3 was purchased from Ionomr Innovations Inc. Electrolytes were prepared with Milli-Q water (18.2 MΩ·cm), and all the chemicals were used as received.

### 2.2 Preparation of Electrodes

#### 2.2.1 Anode (IrO_x_-Coated Titanium Felt)

13 mg iridium­(III) chloride hydrate (99.8%) was dissolved in 1 mL of ethanol, and the solution was sonicated for 1 h. This solution was aged for 1 to 2 days or until it became clear. A 2 cm × 2 cm area of platinized titanium felt was etched in boiling 0.5 M oxalic acid (98%) for 30 min. The etched titanium felt was spray-coated on a hot plate held at 80 °C with the above-prepared solution. The titanium felt coated with IrCl_3_ was then calcinated at 500 °C for 10 min.^10^

#### 2.2.2 Cathode (Cu NP Electrode)

10 mg copper nanoparticle (NP) (25 nm) was added to 10 mL of isopropyl alcohol (IPA). The suspension was sonicated for 30 min to form the catalyst ink. The catalyst ink was sprayed onto the carbon paper GDL using an air spray gun on a hot plate set to 115 °C. The GDL was weighed before and after spray coating to achieve a loading of 0.4 mg cm^–2^, which was maintained throughout the experiment.

### 2.3 Membrane Preparation

#### 2.3.1 Pretreatment of the Nafion 117 Membrane

The Nafion 117 membrane was cut into 2.5 cm × 2.5 cm pieces. The cut pieces were boiled at 80 °C in 3% H_2_O_2_ (prepared by adding 20 mL of 30% H_2_O_2_ to 180 mL of DI water) for 1 h. The Nafion 117 pieces were rinsed in DI water, then boiled in fresh DI water at 80 °C for 2 h. Finally, the Nafion 117 pieces were boiled at 80 °C in 0.5 M H_2_SO_4_ (prepared by adding 5 mL of 98% H_2_SO_4_ to 175 mL of DI water) for 1 h and then rinsed with DI water. The pretreated membrane was always stored in DI water.^16^

#### 2.3.2 Coating of PiperION AEI Film on Nafion 117 Membrane

PiperION (5 wt % solution) was measured in a 1:7 ratio against ethanol on a volumetric basis for the preparation of 200 μL, 400 μL, 600 μL, and 800 μL PiperION anion exchange ionomer layers, respectively. The solution was spray-coated onto a pretreated Nafion 117 membrane (1.5 cm × 1.5 cm portion) on a hot plate set to 90 °C. The film was made sure to have no visible cracks. The prepared AEI film was stored in a 1 M KOH solution.

### 2.4 Electrochemical Measurements

#### 2.4.1 Assembly of Bipolar Membrane Electrode Assembly (BPMEA) Electrolyzer

A rubber gasket with a 1 cm × 1 cm window was placed on the graphite flow channel, attached to the cathode end plate. A 1 cm × 1 cm copper-coated GDE was placed in the rubber gasket window, with the copper surface facing the membrane. The exposed cathode area was covered with an anion-exchange membrane (such as PiperION). Subsequently, Nafion 117 was placed on top of AEM. Finally, a 2 cm × 2 cm iridium-coated titanium felt anode was placed on top of Nafion 117. The electrolyzer was secured by stacking the anodic flow channel on the anode end plate. A torque of 4 Nm was applied to tighten the cell, preventing leaks during operation in the zero-gap configuration.

#### 2.4.2 CO_2_ Reduction in the Membrane Electrode Assembly (MEA) Electrolyzer

The CO_2_ reduction was investigated in a specific MEA electrolyzer that included a GDE cathode, an anion-exchange membrane, a cation-exchange membrane, and an anode made of an iridium-spray-coated titanium felt. A peristaltic pump (Harvard Apparatus P70-7000) controlled the flow rate of the DI water anolyte (5.00 mL/min) across the anode. The dry carbon dioxide feedstock was delivered to the cathode at 20 SCCM via a mass flow controller (Alicat Scientific MC-100SCCM-D). Control experiments were conducted using an anion exchange membrane and a 1 M KOH anolyte. An electrochemical workstation (Solartron EnergyLab XM) controlled the applied cell voltage. The gas products were quantified using gas chromatography (GC, Agilent 7890B), and the liquid products were evaluated using ^1^H nuclear magnetic resonance (NMR) spectroscopy (Bruker AV500).

#### 2.4.3 CO_2_ Crossover Measurement

An extension of the anodic compartment was made by using a PTFE tube (Scheme S2). This ensures that the anodic compartment forms a closed loop with the anolyte recycling. To measure the CO_2_ crossover, the anolyte should be sufficiently saturated with CO_2_, and the CO_2_ crossover was determined by measuring the CO_2_ concentration in the carrier gas stream using GC after steady state was reached. The anodic chamber outlet of the electrolyzer was connected to the inlet of the extended anodic compartment, and the anodic chamber inlet of the electrolyzer was connected to the outlet of the extended cathodic compartment. It was ensured that enough anolyte was filled into the extended anodic chamber before starting the electrolysis. An N_2_ gas stream was used to carry CO_2_ from the anode, and the gas outlet of the extended anodic chamber was connected to the GC.

#### 2.4.4 Gaseous Product Quantification

The Faradaic efficiency (FE) of gas products was calculated using the measured outlet CO_2_ flow rate. A constant stream of argon (10 SCCM) was used as an external standard and evenly mixed with the cell outlet gas stream before injection into the GC column. The standard curve for CO_2_ flow rate was established by a similar method: mixing 10 SCCM Ar gas with a pure CO_2_ stream, with flow rates varying from 5 to 100 SCCM. The concentrations of other gas components were quantified using CO_2_ as the internal standard. The standard GC calibration curves for the other gas components were established using three standard calibration gases: 1000, 2000, and 5000 ppm of H_2_, CO, CH_4_, C_2_H_4_, and C_2_H_6_, respectively, with CO_2_ as the balance gas. The FE of each product was calculated based on the following equation:

$$FE\left(\%\right)=\frac{\frac{A_{x}}{A_{CO_{2}}}}{\frac{A_{x}^{o}}{A_{CO_{2}}^{o}}}\times C_{x}^{o}\times \left[\frac{\left(A_{CO_{2}}/A_{Ar}\right)-b}{a}\times v_{Ar}\right]\times \frac{P}{RT}\times \frac{n_{x}F}{i}$$

where $A_{CO_{2}}$ , *A*_Ar_, and *A_x_* represent the peak area of CO_2_, Ar, and individual gas products in the sample gas; $A_{CO_{2}}^{o}$ and $A_{x}^{o}$ represent the peak area of CO_2_ and a specific gas product in the calibration gas; $C_{x}^{o}$ represents the concentration of the specific gas in the calibration gas; *a* and *b* are the slope and intercept of the linear relationship between the $A_{CO_{2}}/A_{Ar}$ and $v_{CO_{2}}/v_{Ar}$ respectively, from the GC calibration; $v_{CO_{2}}$ is the volumetric flow rate of CO_2_ and *v*_Ar_ is the volumetric flow rate of Ar, which is 10 SCCM; *P* and *T* are the atmospheric pressure and temperature during the test, respectively; *R* is the gas constant; *F* is the Faradaic constant; *n_x_* is the electron transfer number for a specific gas product; and *i* is the total current during the test.^6^

#### 2.4.5 Liquid Product Quantification

25.8 mg of trisodium phosphate was dissolved in 30 mL of D_2_O to prepare a 5 mM solution, which was used as an internal standard. 100 μL of internal standard and 500 μL of sample were pipetted into an NMR tube for analysis. Liquid products were quantified using ^1^H NMR spectroscopy. The concentrations of the liquid products were determined using the NMR peak integral areas and calibration curves. The FE of liquid products was calculated as follows:

$$FE\left(\%\right)=N\times F\times \frac{C\times V}{Q}\times 100\%$$

where *N* is the count of electrons transferred for a required liquid product, *F* is the Faradaic constant, *C* is the concentration of the liquid determined by NMR, *V* is the volume of the electrolyte, and *Q* is the total charge passed during CO_2_ reduction.^6^

## 3 Results and Discussion

### 3.1 Performance Evaluation of BPM Electrolyzers with CEM/AEM Interface

To mitigate CO_2_ crossover in an AEMEA electrolyzer, a BPM configuration was employed by pairing an AEM with a CEM (e.g., a Nafion membrane). In the forward-bias configuration, the AEM faces the cathode while the CEM faces the anode. This arrangement offers dual benefits: the AEM hinders the transport of protons (H^+^) to the cathode, thereby suppressing the HER, while the CEM prevents the crossover of carbonate and bicarbonate ions from the cathodic to the anodic compartments. The Nafion 117 membrane was selected as the CEM due to its robust proton-exchange properties and ion conductivity while effectively inhibiting anion crossover. A thicker Nafion membrane was chosen over a thinner one because it inhibited anion crossover better. Four commercially available AEMs were systematically evaluated for the ECO_2_R performance when paired with Nafion 117 in the BPM configuration. In this BPMEA setup, dry CO_2_ was supplied to the cathode, while deionized (DI) water was fed to the anode. The electrolyzer utilized a copper GDE as the cathode and an IrO_*x*_-coated titanium (Ti) felt as the anode. The BPMEA was configured as follows: Cu GDE/AEM/CEM/IrO_*x*_-Ti felt.

The BPMEA electrolyzer required a higher cell voltage than the AEMEA electrolyzer to achieve similar current densities due to its higher cell resistance and the use of DI water, without a supporting electrolyte, as anolyte, instead of a 1 M KOH electrolyte (Figure S1). Over a range of potentials from 3.6 to 4.0 V, the AEM#4 (PiperION, 80 μm)/Nafion 117 configuration consistently performed at the highest current density (Figure 1a). AEM#4/Nafion 117 exhibited the lowest FE of H_2_, ranging between 20% and 30% at lower cell voltages from 3.6 to 4.2 V (Figure 1b). However, an increase in cell voltage from 4.2 to 4.6 V corresponded with a more pronounced increase in the HER rate. In contrast, as the cell voltage increases, a decrease in selectivity for C_1_ products (CO and HCOO^–^) was observed for all BPMEAs (Figure 1c). AEM#4/Nafion 117 showed the lowest selectivity for C_1_ products. Conversely, the AEM#4/Nafion 117 demonstrated the highest FE for C_2+_ products, including C_2_H_4_, C_2_H_5_OH, CH_3_COO^–^, and CH_3_CH_2_CH_2_OH, with a maximum FE of ∼62% at 4.0 V (Figure 1d). The lowest C_2+_ FE is obtained with AEM#3/Nafion 117, and moderate and similar C_2+_ FE is observed for both AEM#1/Nafion 117 and AEM#2/Nafion 117.

**Figure 1:**
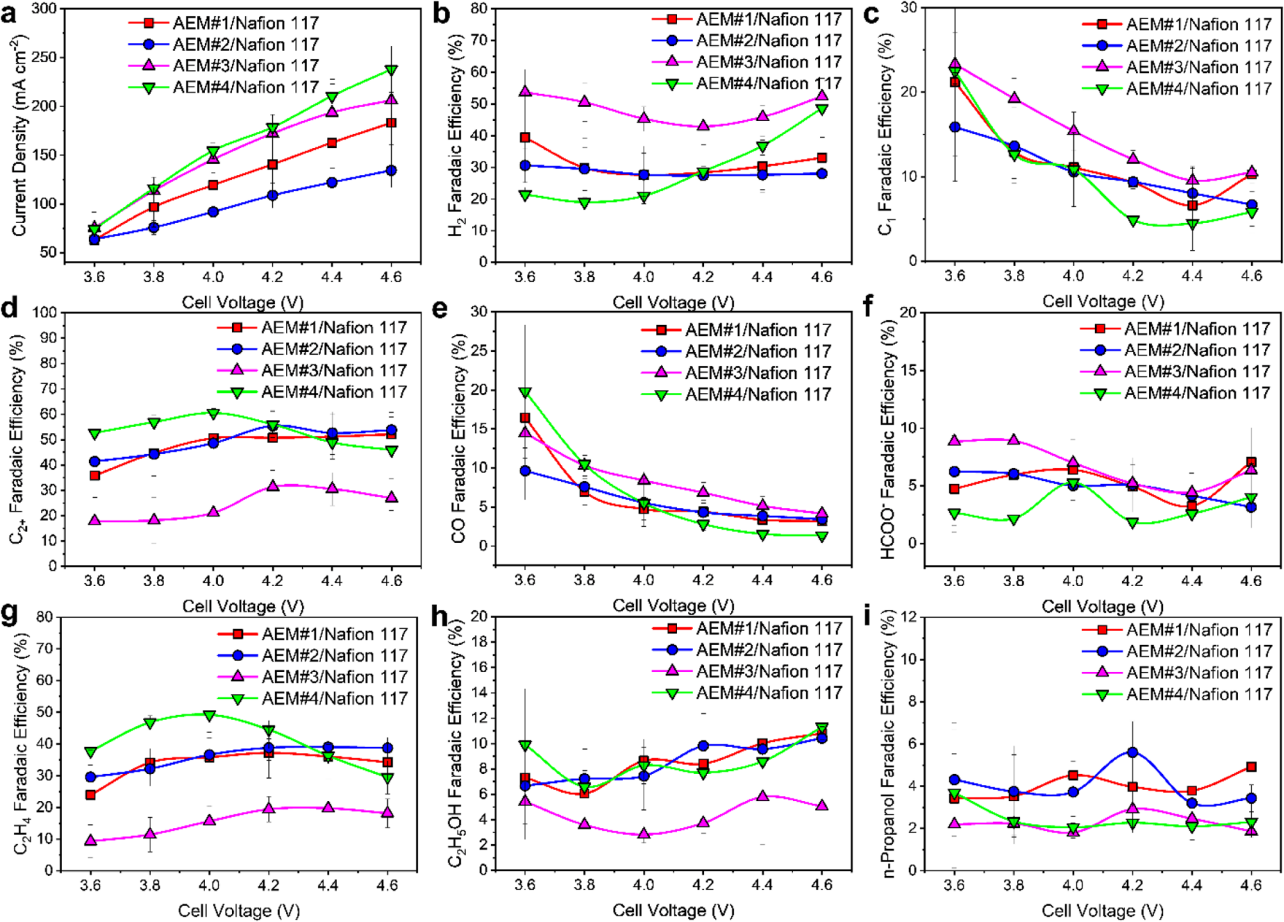
ECO_2_R in forward bias BPMEA cells with dry CO_2_ fed to the cathode and DI water supplied to the anode. (a) Overall current density, (b–i) Faradaic efficiency of (b) H_2_ and (c) C_1_ products (CO and HCOO^–^), and (d) C_2+_ products (C_2_H_4_, C_2_H_5_OH, CH_3_COO^–^, and n-C_3_H_7_OH), (e) CO, (f) HCOO^–^, (g) C_2_H_4_, (h) C_2_H_5_OH, and (i) n-C_3_H_7_OH as a function of cell voltage. AEM (cation): AEM#1 (benzimidazolium), AEM#2 (quaternary ammonium), AEM#3 (imidazolium), and AEM#4 (piperidinium).

ECO_2_R is proven to take place exclusively in the presence of cations in the electrolyte.^17^ In pure water that this BPM electrolyzer utilizes, in the absence of alkali metal cations, organic cations embedded within the AEMs act as a kind of pseudo-outer Helmholtz plane.^18^ Hence, the organic cations are likely able to stabilize intermediates via dipole-local electric field interactions, enabling CO_2_ activation and C–C coupling to C_2+_ products.^14,19,20^ The cation-derived local electric field stabilizes the dipolar C_2_ intermediates such as *OCCO and *OCCHO and thereby accelerates C–C coupling.^19^ Hence, the C_2+_ product selectivity of polymer cations can, in general, be correlated with the Bader/positive charge on the N atom of cations.^21^ The above-mentioned four AEMs can be grouped into two groups, two AEMs in each group, based on their Bader/positive charge on N atom(s) of their cations. Quaternary ammonium cation (AEM#2) and piperidinium cation (PiperION/AEM#4), where the positive charge is located/distributed on their single aliphatic N atom with no aromatic conjugation, result in both AEMs having similar or higher Bader/positive charges on the N atom(s) of their cations, leading to a higher local electric field strength at the electrode-membrane interface.^22^ Further, for benzimidazolium cation (AEM#1) and imidazolium cation (AEM#3), the positive charge on the N atoms is in continuous conjugation within an aromatic ring, resulting in charge delocalization over two N atoms, which can have similar and lower Bader/positive charges on the N atom(s) of their cations, leading to lower electric field strength compared to the other group (AEM#2 and AEM#4).^23^ Moreover, factors such as ion-exchange capacity (IEC) influence CO_2_R selectivity as it corresponds to the concentration/density of cationic groups in the membrane. The higher the cation density/concentration, the higher the electric field and the better the stabilization of CO_2_R intermediates. The performance/C_2+_ product selectivity depends on both the strength of the single cation (Bader/positive charge on the N atom) in stabilizing the CO_2_R intermediates and the number of cations per unit area (IEC), as both contribute to increased electric field strength. Among AEM#1/Nafion 117 (benzimidazolium cation) and AEM#3/Nafion 117 (imidazolium cation), which possess similar Bader charges, the least C_2+_ FE is obtained on AEM#3/Nafion 117 due to its low IEC of 1.1 mequiv g^–1^ compared to the IEC of AEM#1 (2.3–2.6 mequiv g^–1^). Among AEM#2/Nafion 117 (quaternary ammonium cation) and PiperION/Nafion 117 (piperidinium cation), which possess similar Bader charges, the highest C_2+_ FE is obtained on PiperION/Nafion 117 due to its high IEC of 2.0–2.3 mequiv g^–1^ compared to the IEC of AEM#2 (1.6–2.1 mequiv g^–1^) (Table S2). Further, AEM#1/Nafion 117 (benzimidazolium cation) and AEM#2/Nafion 117 (quaternary ammonium cation) show similar selectivity for C_2+_ products because of a trade-off between the effects of the nature of the cation and the cation density, i.e., AEM#1 possesses a lower Bader charge on its N atom of its cation and high IEC, and AEM#2 possesses a high Bader charge on the N atom of its cation and low IEC. Therefore, the higher IEC/charge density of AEM#1 compensates for the inadequate cationic effect, equalizing C_2+_ selectivity with AEM#2. From the above discussion, it can be concluded that the highest C_2+_ FE is obtained with AEM#4/Nafion 117 due to the high Bader charge on the N atom of its cation and its high IEC. Conversely, the lowest C_2+_ FE is obtained with AEM#3/Nafion 117 due to a low Bader charge on the N atom of its cation and a low IEC. Further, the combined effect of Bader charge and IEC for AEM#1/Nafion 117 and AEM#2/Nafion 117 is the same, less compared to AEM#4, and high compared to AEM#3, resulting in equal and moderate C_2+_ selectivity (Scheme S3).

We analyzed the AEM dependency of FE of specific products. FEs of both CO and HCOO^–^ followed a similar trend, i.e., AEM#3 is highest, AEM#4 is lowest, and AEM#1 and AEM#2 are moderate for all BPM combinations (Figure 1e, f). The decrease in CO FE with increasing potential is because CO dimerization occurs more than CO desorption as *CO coverage and local pH increase. The FE of C_2_H_4_ is the highest for AEM#4, with a C_2_H_4_ FE of ∼50% at a cell voltage of 4 V (Figure 1g). But, surprisingly, AEM#4 showed low ethanol selectivity, comparable to that of AEM#1 and #2. The reduced FE for ethanol on AEM#4 can be rationalized by considering the interplay between cation structure, interfacial water structure, and the divergence between C_2_H_4_ and ethanol formation from a common selectivity-determining intermediate (SDI). The SDI, most likely a carbon-bound *HCCOH intermediate, can undergo dehydration mediated by cation-associated H_2_O to form C_2_H_4_, or hydrogenation by adsorbed *H to produce ethanol.^24,25^ Water molecules coordinated with cations are typically more weakly hydrogen-bonded than bulk water, as electrostatic interactions with the cation disrupt intermolecular hydrogen-bonding networks.^26^ As a result, cation-associated H_2_O can more readily reorient, enhance water dissociation, and protonate the hydroxyl group of *HCCOH, facilitating dehydration of the SDI.^27^ Owing to its higher cation charge density, AEM#4 likely contains a greater population of cation-associated, weakly hydrogen-bonded water molecules, which accelerates dehydration of the SDI toward C_2_H_4_ formation and consequently lowers ethanol selectivity. AEM#3 shows the least ethanol selectivity due to the inferior overall C_2+_ selectivity (Figure 1h). n-Propanol has shown no specific trend, which may be due to its low FE (Figure 1i).

It is intuitive that the BPMEA configuration inhibits the CO_2_ crossover compared to the AEMEA configuration due to the regenerative nature of the membrane interface, which converts carbonate ions transported from the cathode to CO_2_ by protons transported from the anode. BPMEA configuration also enhances stability by inhibiting salt precipitation, thanks to its use of DI water as an electrolyte. However, commercial membranes lack porosity, which is necessary for recycling regenerated CO_2_ and removing regenerated water at the membrane interface. The use of nonporous AEM in BPM configurations can lead to membrane delamination, limiting the system’s stability. To address this issue, we coated a microporous AEI layer onto the Nafion 117 membrane.

### 3.2 Modified BPM Design with AEIPL/CEM Interface

Since PiperION AEM in combination with Nafion 117 showed high overall activity, low HER FE, and high C_2_H_4_ FE compared to the other three AEMs, we intend to fabricate PiperION AEIPL with the required porosity and thickness on the Nafion 117 membrane. An optimal AEIPL thickness is needed to balance low HER activity (suppressed by thicker films) and low cell voltage (favored by thinner films). To identify this optimum, PiperION AEI films of 20, 35, 50, and 70 μm (estimated from SEM) were prepared by spray-coating 200, 400, 600, and 800 μL of a 5 wt % PiperION and ethanol (1:7 v/v) solution onto a Nafion membrane, respectively. Top-view and cross-sectional scanning electron microscopy (SEM) images reveal that the 70 μm-thick AEIPL has a rough surface but is free of cracks (Figure 2a–c). Additionally, Figure 2d indicates that the AEI film exhibits a degree of porosity, which is significant for the transport of both water and gas. As Nafion is composed of elements C, F, O, and S, and PiperION is composed of elements C, F, O, and N, elemental analysis/EDS mapping of S and N can clearly distinguish between membranes. We have therefore performed elemental mapping of these elements. Cross-sectional scanning electron microscopy (SEM) of the PiperION AEIPL/Nafion 117 reveals a well-defined, intact interface (Figure S2a), which is further substantiated by phase mapping analysis (Figure S2b). Concurrently, EDS elemental mapping clearly delineates the interface, resolving distinct spatial accumulations of N and S signals localized within the respective PiperION and Nafion domains (Figure S2e).

**Figure 2:**
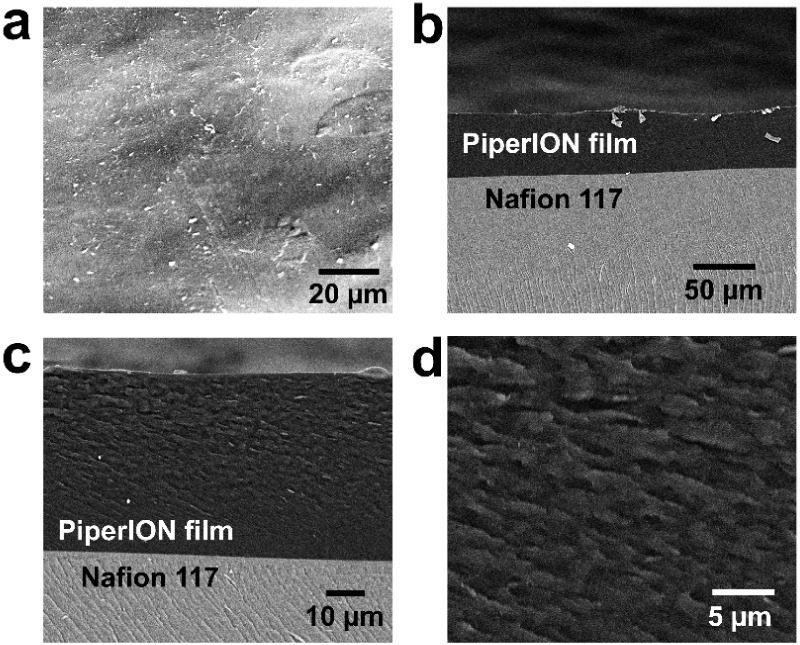
SEM image of 70 μm-thick PiperION AEIPL onto Nafion 117 membrane. (a) Top view of PiperION AEIPL, (b,c) low-magnification cross-sectional view of interface between PiperION AEIPL and Nafion 117 membrane, (d) high-magnification cross-sectional view of PiperION AEIPL indicating microporosity.

The current density decreases as the thickness of the PiperION AEIPL increases, due to increased resistance (Figure 3a). At a lower cell voltage of 3.6 V, the thinnest PiperION AEI coating (20 μm) exhibited the highest H_2_ FE of 87%. At an optimum cell voltage of 4.0 V, the 20 μm PiperION AEI film exhibited the lowest H_2_ FE compared to that of the 70 μm film (Figure 3b). As the cell voltage increases, the thinnest PiperION AEI coating at 20 μm exhibited the highest CO FE of 22% (Figure 3c) and C_1_ product FE of 26% (Figure 3d) among all other thicknesses. These results contrast with our prediction that the thinnest film imposes the highest H^+^ concentration at the electrode interface and favors the highest HER over the entire potential range. This may be because CO_2_R is kinetically favored and competes with HER at higher potentials. Similarly, the same BPM configuration exhibited the lowest FE of 21% for C_2_H_4_ (Figure 3e) and 26% for C_2+_ products (Figure 3f) among all other thicknesses. As thickness increased, C_2+_ product selectivity increased, and the 70 μm AEI coating demonstrated a C_2+_ FE of 51%, approaching that of the commercial PiperION AEM/Nafion 117 BPM. The increase in overall AEI and CEM thickness reduces H^+^ conductance and water transport, thereby impacting selectivity.^28^ Lower H^+^ conductance results in higher pH near the catalyst surface, which favors C–C coupling in C_2+_ products.^29^ Further, lower water uptake/higher hydrophobicity also favors C_2+_ products.^30^

**Figure 3:**
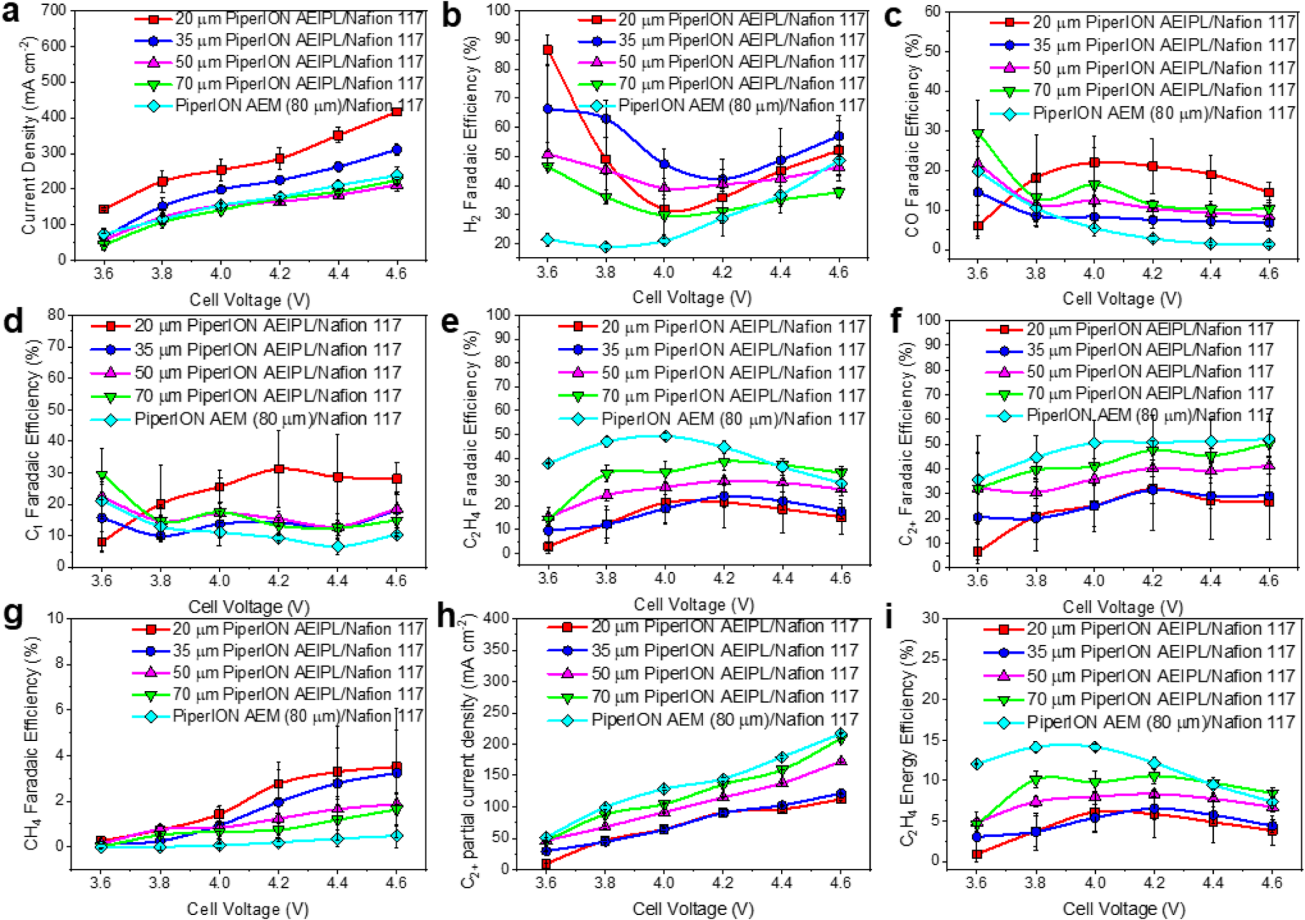
PiperION AEI coating at varying thicknesses on Nafion 117 membrane (ECO_2_R in DI water). (a) Total current density, (b) H_2_ Faradaic efficiency, (c) CO Faradaic efficiency, (d) C_2_H_4_ Faradaic efficiency, (e) Faradaic efficiency of C_1_ products (CO, CH_4_, and HCOO^–^), (f) Faradaic efficiency of C_2+_ products (C_2_H_4_, C_2_H_5_OH, CH_3_COO^–^, and n-C_3_H_7_OH), (g) CH_4_ Faradaic efficiency, (h) C_2+_ partial current density, and (i) C_2_H_4_ energy efficiency as a function of potential.

The thin AEI film allows proton influx, thereby lowering the pH at the cathode. The lower pH or high H^+^ concentration at the cathode favors HER at lower overpotentials and methane production at higher overpotentials (Figure 3g).^29^ The increased thickness of the AEI film prevents proton flux, maintaining a basic environment at the cathode. Higher pH favors C–C coupling and C_2+_ product formation due to a higher local OH^–^ concentration, as detailed earlier.^29^ At a cell potential of 4.2 V, PiperION AEM/Nafion 117 BPM showed C_1_ products FE of 13%, H_2_ FE of 30%, and C_2_H_4_ FE of 39%, and 70 μm PiperION AEI coating showed selectivity close to this, i.e., C_1_ products FE of 10%, H_2_ FE of 29%, and C_2_H_4_ FE of 44% (Figure 3b, d). As CO is an important intermediate for the formation of C_1_ and C_2+_ products, CO as a product (in desorbed form) should be moderate to produce hydrocarbons or oxygenates. Figure 3c shows that a 70 μm-thick PiperION AEI film showed a moderate FE of 14% for CO. From these results, it can be concluded that the 70 μm-thick PiperION AEI film’s performance in terms of activity, ethylene, and ethanol selectivity is most comparable with commercial PiperION AEM (Figure S3a). Moreover, both the C_2+_ partial current density (Figure 3h) and C_2_H_4_ energy efficiency (Figure 3i) plots suggest that 70 μm AEIPL matches the performance of the standard PiperION. Hence, the 70 μm-thick PiperION AEI film was chosen for the optimal BPM cell configuration due to its combination of high C_2+_ selectivity and low HER. The crossover performance of the AEIPL BPM electrolyzer was compared to that of the standard PiperION MEA/Nafion 117 BPM electrolyzers. These results suggest that a thickness of 70 μm is best.

### 3.3 CO_2_ Crossover Analysis of AEIPL/CEM BPMEA Electrolyzer

The CO_2_ crossover in the 70 μm PiperION AEIPL/Nafion 117 CEM BPMEA electrolyzer with DI water as the anolyte was evaluated under two conditions: (1) a constant CO_2_ flow rate of 20 SCCM with varying current densities, and (2) a constant current density of 150 mA cm^–2^ with varying CO_2_ feed rates. For comparison, two control configurations were also tested: a PiperION AEMEA electrolyzer with 1 M KOH anolyte and a PiperION AEM/Nafion 117 CEM BPMEA electrolyzer with DI water anolyte.

Under condition (1), Figure 4a shows that the PiperION AEMEA in KOH exhibited significant CO_2_ loss up to 2.5 SCCM (12.5%) at 300 mA cm^–2^ due to increased carbonate/bicarbonate crossover at higher current densities. In contrast, both BPMEA systems (PiperION AEM & Nafion 117 CEM and PiperION AEIPL/Nafion 117 CEM) maintained minimal and stable CO_2_ crossover across the current density range (50–300 mA cm^–2^). The 70 μm PiperION AEIPL slightly outperformed the PiperION AEM in mitigating crossover (Figure 4a). Although CO_2_ crossover typically increases with current density, the BPMEA systems showed little variation, suggesting effective in situ CO_2_ regeneration by the BPMs. Correspondingly, the PiperION AEMEA cell showed the lowest amount of unreacted CO_2_ at the cathode outlet (Figure S4a, b).

**Figure 4:**
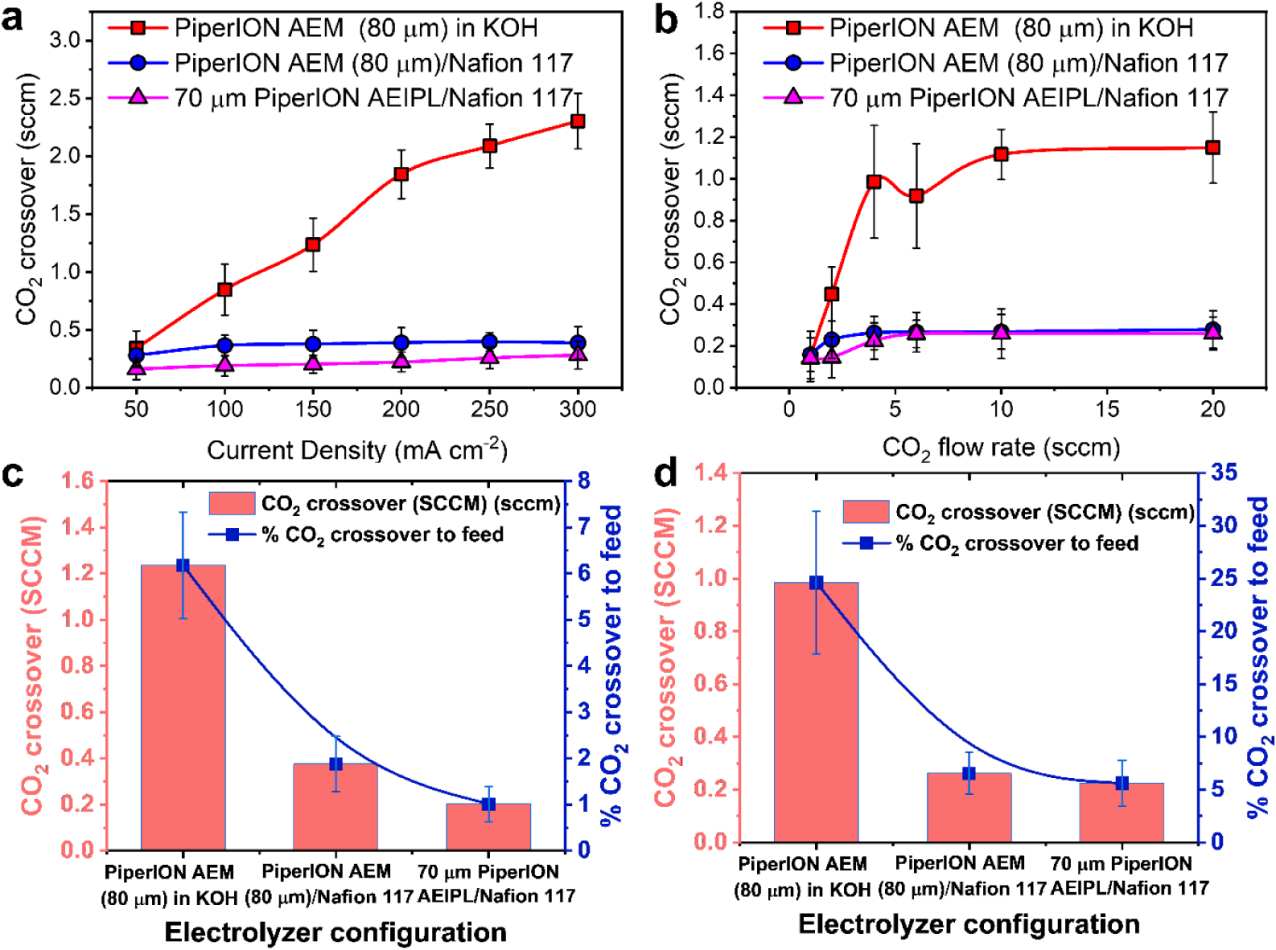
Comparison of CO_2_ crossover for electrolyzer configurations. (a) Feed flow rate at 20 SCCM by varying current densities, (b) current density of 150 mA cm^–2^ by varying feed rates. CO_2_ crossover and CO_2_ crossover-to-feed ratio calculated at (c) 150 mA cm^–2^ and 20 SCCM from varying current density plot (a) and (d) 150 mA cm^–2^ and 4 SCCM from varying feed rate plot (b).

At 150 mA cm^–2^ and a CO_2_ feed rate of 20 SCCM, the PiperION AEMEA allowed 1.2 SCCM (6%) of CO_2_ crossover (Figure 4a, c). Incorporating a PiperION AEM/Nafion 117 CEM BPM reduced the loss to 0.4 SCCM. The 70 μm-thick PiperION AEI/Nafion 117 CEM BPMEA further improved performance, cutting crossover to 0.2 SCCM. This configuration reduced CO_2_ crossover from 6% (PiperION AEMEA) to just 1% (Figure 4c).

Under condition (2), CO_2_ feed rates from 1 to 20 SCCM were tested at a constant 150 mA cm^–2^ to study the feed rate impact on the CO_2_ crossover (Figure 4b). The PiperION AEMEA cell showed a sharp increase in CO_2_ crossover at CO_2_ feed rates up to 4 SCCM, followed by a more gradual rise at higher flow rates. This indicates that at low flow rates, crossover is limited by the CO_2_ feed rate (or by mass transport, which limits neutralization with OH^–^), whereas at higher flow rates it becomes current-limited, consistent with observations in Figure 4a. In contrast, both BPMEA configurations maintained low crossover even as the feed rate increased. Notably, the PiperION AEI exhibited better crossover suppression than the PiperION AEM at lower feed rates.

At a current density of 150 mA cm^–2^ and a CO_2_ feed rate of 4 SCCM (i.e., near stoichiometric supply), the PiperION AEMEA allowed 1.0 SCCM of CO_2_ crossover (Figure 4)­d. Introducing a PiperION AEM/Nafion 117 CEM BPM reduced the loss to 0.26 SCCM. The 70 μm PiperION AEIPL/Nafion 117 CEM BPM further minimized crossover to 0.20 SCCM. This configuration reduced CO_2_ crossover from 25% (PiperION AEMEA) to 5%, attributed to enhanced CO_2_ regeneration and efficient transport through the microporous structure in the AEIPL.

### 3.4 Stability Analysis of AEIPL/CEM BPMEA Electrolyzer

To further establish the merit of the BPM design, we assessed its long-term operational stability relative to standard BPM and AEMEA electrolyzers. The PiperION AEMEA electrolyzer using 1 M KOH anolyte was tested at a constant current density of 200 mA cm^–2^. As shown in Figure 5a, b, the initial cell voltage was 2.63 V, and the FE of C_2_H_4_ and H_2_ was 50% and 18%, respectively. During the first hour, the FE of C_2_H_4_ remained stable, but the FE of H_2_ increased significantly. Over time, the FE of C_2_H_4_ gradually declined while the H_2_ FE continued to rise. After 3.5 h, gas flow ceased due to pressure buildup in the supply line, caused by the formation of potassium bicarbonate salts in the gas diffusion layer (GDL) and cathode flow field. This “salting-out” resulted from K^+^ ions migrating from the anode and reacting with carbonate/bicarbonate species formed during CO_2_ electrolysis.^31^ To restore gas flow, DI water was injected into the cathode using a syringe to flush out the precipitated salts. While this intervention resumed flow, it did not fully recover catalytic selectivity. C_2_H_4_ selectivity decreased further, while HER activity increased. After 8 h of electrolysis, H_2_ FE increased to 84%, while C_2_H_4_ FE dropped to 10%. A second salting-out event was observed, and although flushing again removed the salts, no improvement in ethylene selectivity was achieved. A slight decrease in cell potential of 100 mV was observed during 8 h of electrolysis, especially after water flushing, which may be attributed to the facile kinetics of HER compared to ECO_2_R (Figure 5a).

**Figure 5:**
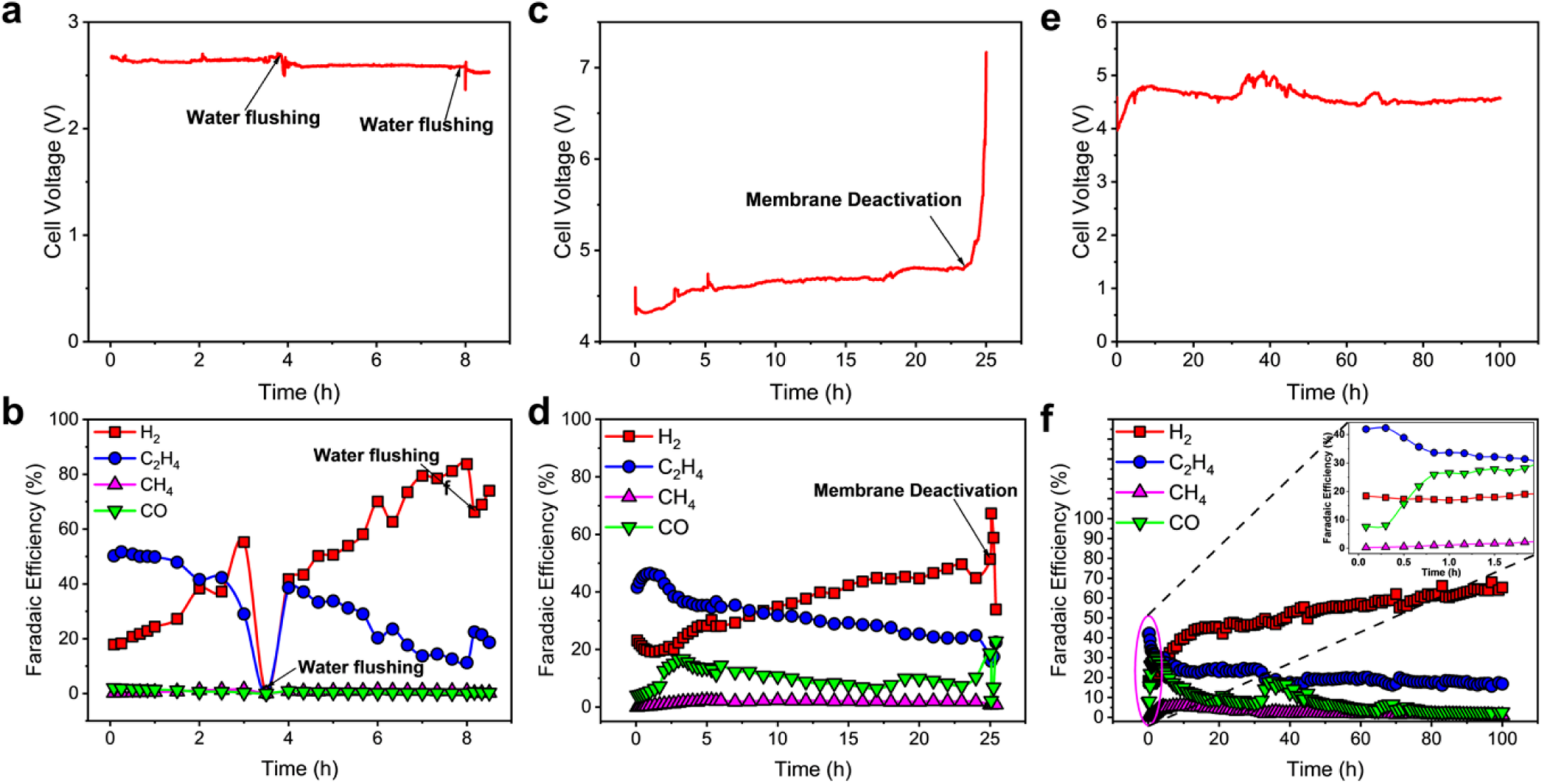
Stability analysis at the current density of 200 mA cm^–2^. (a) Cell voltage vs time and (b) Faradaic efficiency vs time of PiperION AEMEA with 1 M KOH anolyte. (c) Cell voltage vs time and (d) Faradaic efficiency vs time of PiperION AEM/Nafion 117 BPM cell with DI water anolyte. (e) Cell voltage vs time and (f) Faradaic efficiency vs time of 70 μm-thick PiperION AEIPL/Nafion 117 BPM with DI water anolyte.

The PiperION AEM/Nafion 117 CEM BPM electrolyzer, operated with DI water and a current density of 200 mA cm^–2^, exhibited increasing cell voltage from 4.3 V to 4.8 V over 23 h (Figure 5c, d). This increase in voltage was due to increased in cell resistance caused by the buildup of CO_2_ and water at the Nafion 117-AEM interface, as there is no path for these species to escape. The higher operating voltage of the BPM cell relative to the AEMEA is consistent with the higher ionic resistance of BPMs, which depends on membrane thickness and the absence of a supporting electrolyte.^11^ Initial FE values of 46% for C_2_H_4_ and 20% for H_2_ were observed. As electrolysis continued, C_2_H_4_ FE gradually decreased, while H_2_ FE increased. An abrupt rise in H_2_ FE occurs at 25 h, resulting in H_2_ FE exceeding 70%. At this point, the cell voltage abruptly rose to 7.1 V, rendering the system inoperable. This failure was caused by delamination at the AEM/CEM interface due to mechanical stress from gas buildup.^32,33^ The gas (CO_2_) was formed by the reaction between carbonate/bicarbonate ions and protons at the AEM/CEM interface.^5,6,34,35^ The delamination, resulting in a loss of the zero-gap configuration, was irreversible and marked the membranes’ deactivation.

In contrast, the 70 μm PiperION AEIPL/Nafion 117 CEM BPM electrolyzer demonstrated better long-term stability. Operated under identical conditions (200 mA cm^–2^, DI water), it began with a cell voltage of 4.0 V, 42% C_2_H_4_ FE, and 15% H_2_ FE (Figure 5e, f). The cell voltage initially increased from 4.0 to 4.5 V within the first 10 h. Afterward, throughout the next ∼100 h of continuous operation, the cell voltage remained 4.5 V. C_2_H_4_ FE decreased from 42% to 24% in the first 8 h of operation and remained stable for 31 h. After that, it decreased again to 17% and remained stable until 100 h. The rate of H_2_ FE was lower for the AEIPL-based BPM than for the AEM-based BPM. Enhanced durability is attributed to the microporous structure of the AEI film (Figure 2d), which facilitates CO_2_ regeneration and allows water and gas to diffuse away from the AEI/CEM interface. This prevents pressure buildup and mechanical failure. However, the selectivity of C_2_H_4_ still degraded over time. To understand the reason behind the degradation of C_2_H_4_ selectivity, SEM analysis of the GDE was performed before and after 100 h of electrolysis, revealing no change (Figure S5). Although membrane analysis is not performed, the same membrane is used after stability analysis with the new dry copper GDE, and similar results are obtained as with the new one. Further, water flooding is clearly visible at the outlet tube of the electrolyzer. These results indicate that the selectivity loss of C_2_H_4_ is mainly due to water flooding, rather than to the copper-GDE or the membrane. We have carried out long-term EIS measurements for PiperION AEIPL/Nafion 117 and PiperION AEM/Nafion 117 BPM to analyze the effect of interfacial resistance and charge transfer resistance in stability performances of both BPMs (Figure S6a, b), and the EIS data was simulated by a modified Randles circuit (Figure S6c). The ohmic resistance (R_s_) and the charge transfer resistance (R_ct_) of the PiperION AEIPL/Nafion 117 remained nearly constant throughout the 100-h stability test, in agreement with a stable membrane interface (Table S3), and catalyst/electrolyte interface and electrode kinetics, respectively. The R_s_ of the PiperION AEM/Nafion 117 BPM also remained constant around 1.028 Ω until 24 h (Table S4). But R_ct_ increased rapidly for PiperION AEM/Nafion 117 after 24 h. AEM/cathode catalyst layer and Nafion/anode catalyst layer delamination directly creates interfacial voids, which immediately increase interfacial charge-transfer resistance. This is the most direct degradation mode, leading to an increase in charge transfer resistance. Nevertheless, the AEI-based BPM electrolyzer achieved at least four times the stability of the standard BPM system and over 30 times the stability of the AEMEA electrolyzer.

## 4 Conclusions

In summary, the PiperION membrane achieves C_2_H_4_ and C_2+_ FE comparable to those obtained with alkali-metal cations, which we attribute to the enhanced interfacial electric field generated by membrane-embedded nonconjugated piperidinium cations resulting from the combined effect of both high Bader charge on the N atom of its cation and high IEC. Leveraging the performance of this membrane, a porous CO_2_ regenerative film was coated by using PiperION ionomer over Nafion 117 to fabricate an AEI-BPM. The resulting AEI-based BPM reduced the CO_2_ crossover from 25% to 5% at a current density of 150 mA cm^–2^ and a CO_2_ feed flow rate of 4 SCCM. Moreover, the AEI-BPM maintained a stable cell voltage over 100 h of continuous operation. While the BPM configuration successfully prevented salt precipitation, long-term performance was constrained by water flooding within the gas-diffusion channels. Effective water management thus emerges as the next critical challenge to further extend stability and enable full-scale deployment of CO_2_ electro-reduction systems. Furthermore, the cation structure and IEC can serve as new design parameters for the fabrication of anion-exchange membranes for electrochemical CO_2_ reduction in systems that operate with cation-free electrolytes.

## Supplementary Material



## Notes

Summary table comparing with previous works, properties and cation structures of commercial anion exchange membranes, Faradaic efficiency of products in AEMEA, and PiperION AEI coating at varying thicknesses on Nafion 117 membrane (PDF)
