# Supplementary material for: Influence of CO2‑Regenerative Film Properties in Enhancing C2+ Products Selectivity While Mitigating CO2 Crossover
Source: Energy Fuels. 2026 Jun 29;40(27):14654–63. doi: 10.1021/acs.energyfuels.6c01357 (PMC13359101; doi:10.1021/acs.energyfuels.6c01357)
Supplement: Supplementary file 1 [file ef6c01357_si_001.pdf]

Supporting Information

Influence of CO_2-Regenerative Film Properties in Enhancing C_{2+} Products Selectivity while Mitigating CO_2 Crossover

Ashok Kumar Ummireddi,[1,2]* Ananya Vasudevan,[1] Jithu Raj,[1] Mohammad Fahim Yasir,[1] and Jingjie Wu[1]*

[1]Department of Chemical and Environmental Engineering, University of Cincinnati, OH 45221, USA

[2]Department of Chemical Engineering, Indian Institute of Technology Dharwad, KA 580011, India

*Corresponding Author: wu2jj@ucmail.uc.edu, ashok@iitdh.ac.in

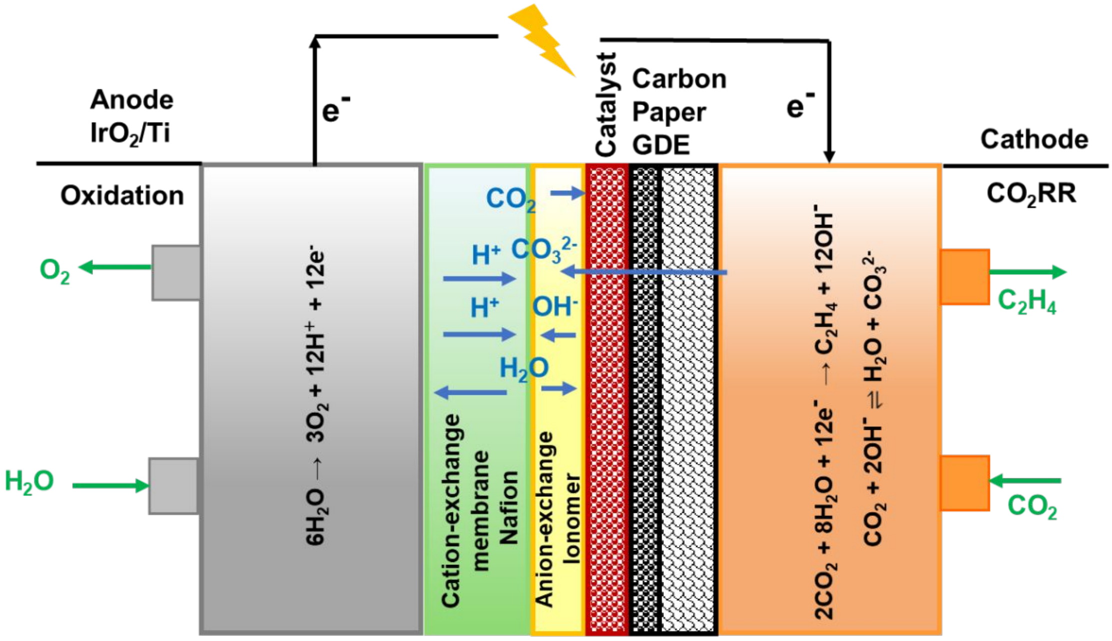

Scheme S1. Schematic of bipolar membrane electrode assembly cell (BPMEA) configuration with anion exchange ionomer film coating over Nafion 117 membrane for electrochemical CO_2 reduction.

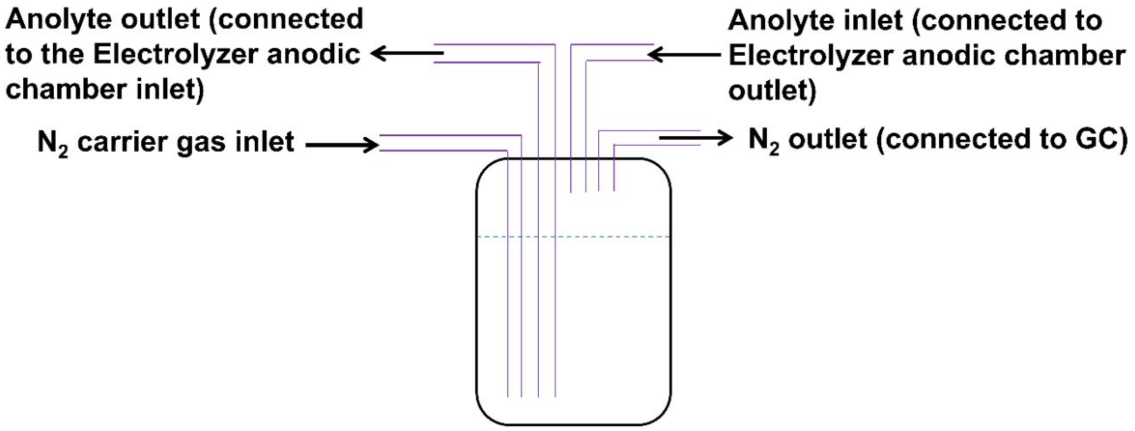

Scheme S2. The schematic of the extended anodic compartment for CO_2 crossover quantification.

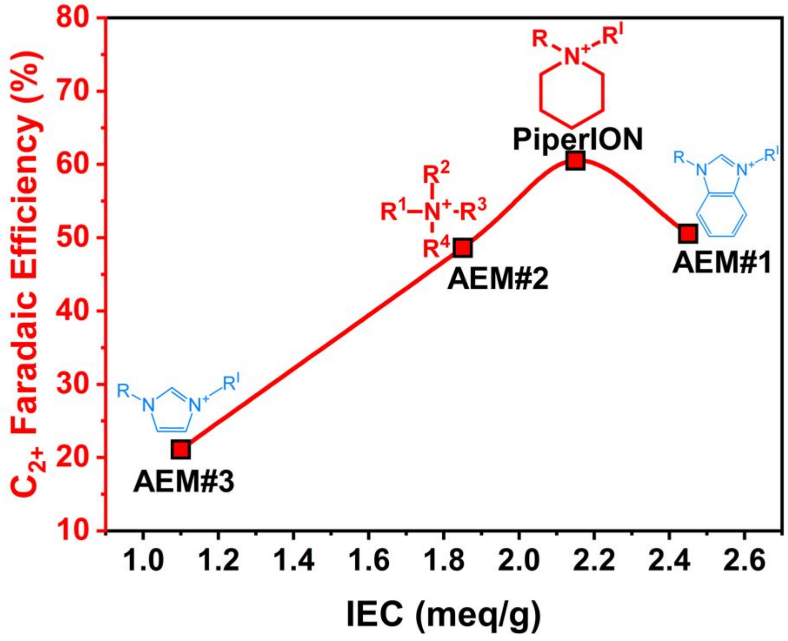

Scheme S3. Influence of the cationic nature and density (IEC) of the CO_2 regenerative film on CO_2R selectivity toward C_{2+} products.

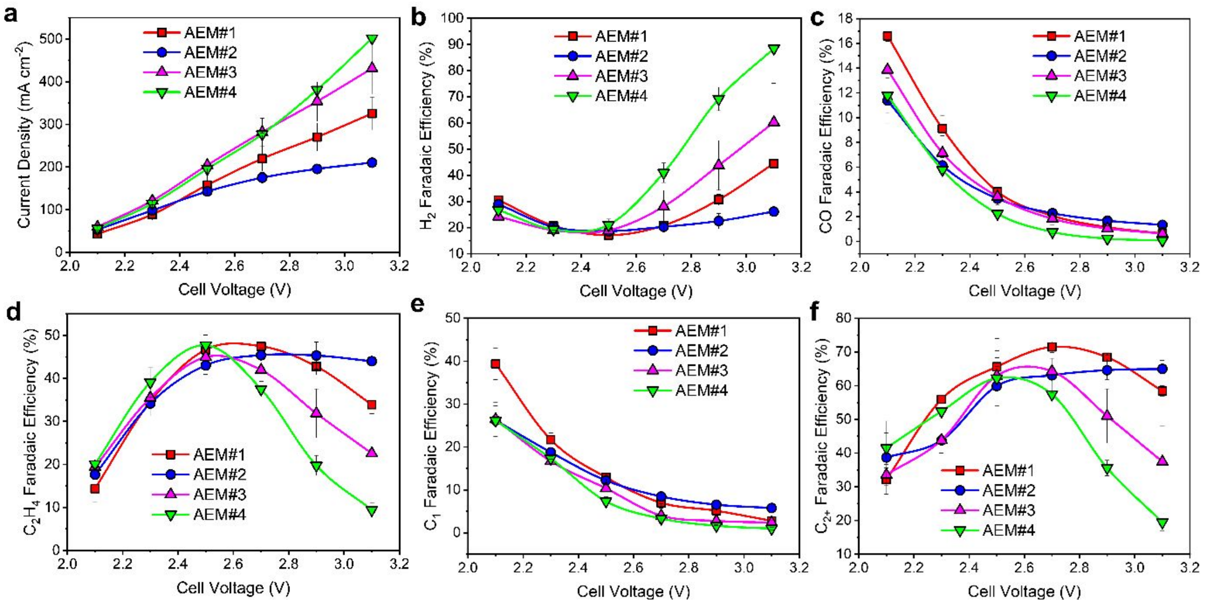

Figure S1. AEMEA (ECO_2R in 1 M KOH); (a) Total current density, (b) H_2 Faradaic efficiency, c) CO Faradaic efficiency, (d) C_2H_4 Faradaic efficiency, (e) Faradaic efficiency of C_1 products (CO, CH_4, and $HCOO^-$), and (f) Faradaic efficiency of C_{2+} products (C_2H_4, C_2H_5OH, CH_3COO^-, and n-C_3H_7OH) as a function of potential.

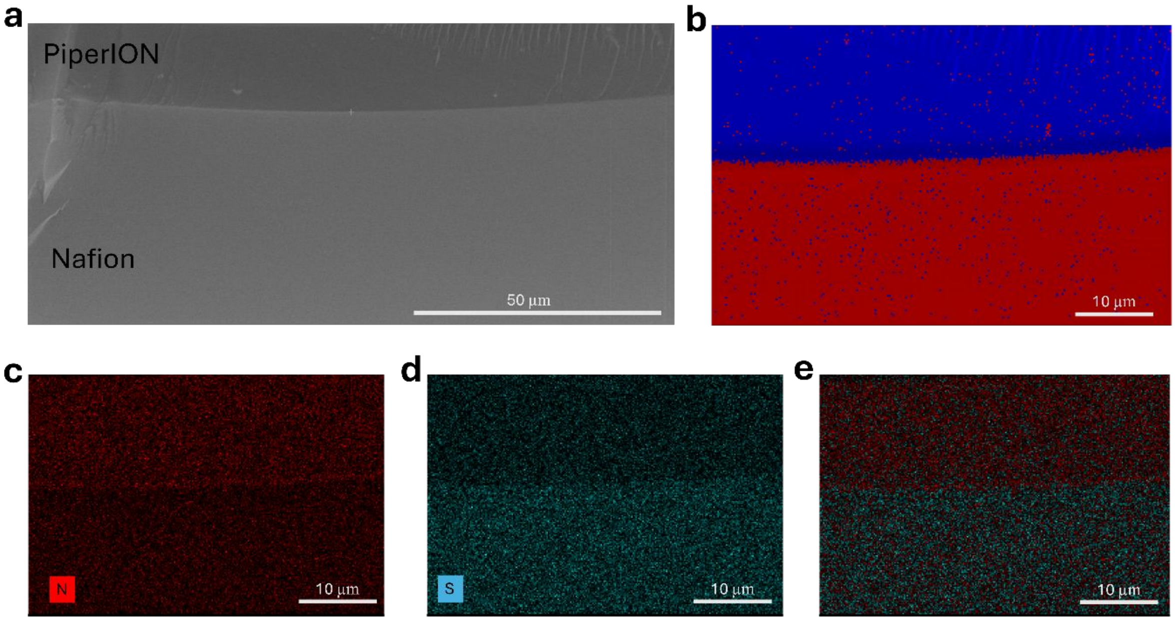

Figure S2. SEM-EDS analysis of the cross-section of the PiperION AEIPL/Nafion 117 interface. (a) SEM cross-section image, (b) phase map, (c, d) EDS elemental mapping of N (c) and S (d), and (e) overlap of EDS mapping of N and S.

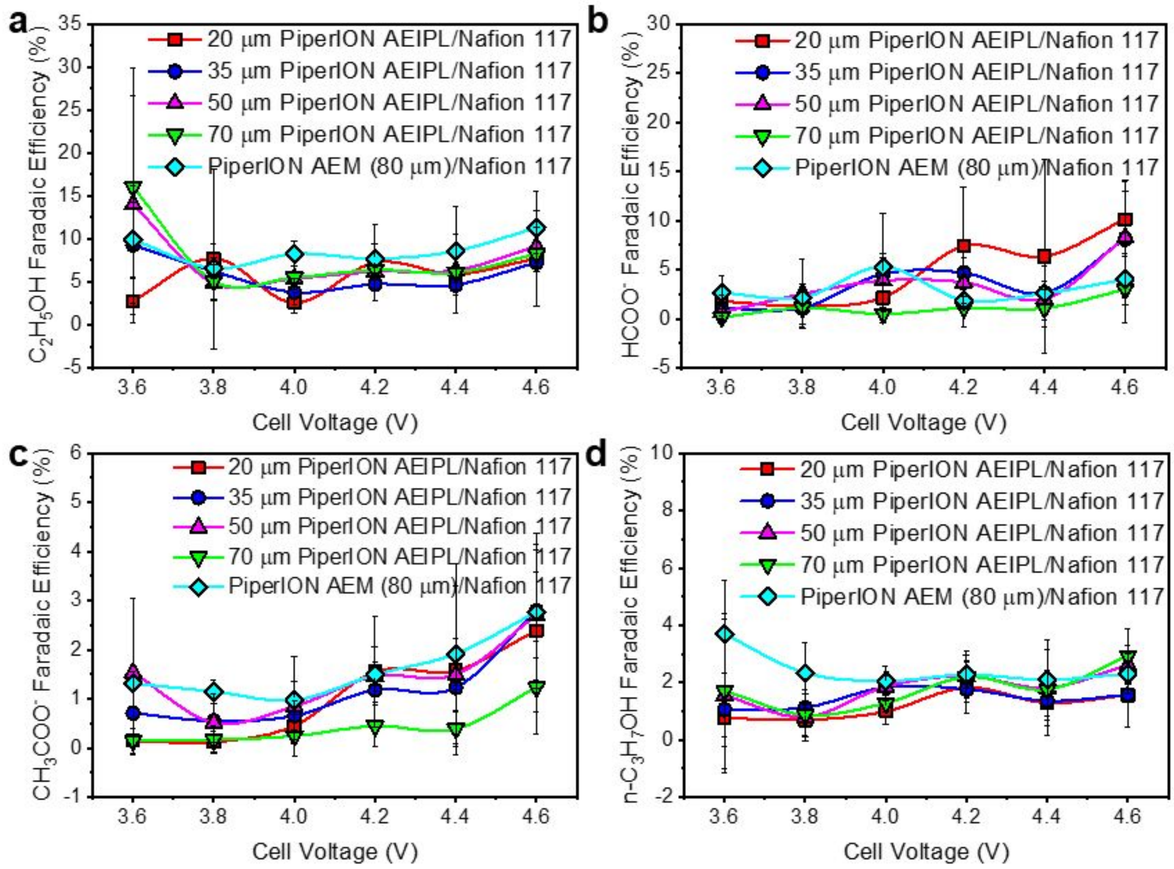

Figure S3. PiperION AEI coating at varying thicknesses on Nafion 117 membrane (ECO_2R in DI water); (a) C_2H_5OH Faradaic efficiency, (b) $HCOO^-$ Faradaic efficiency, (c) CH_3COO^- Faradaic efficiency, and (d) n-C_3H_7OH Faradaic efficiency as a function of potential.

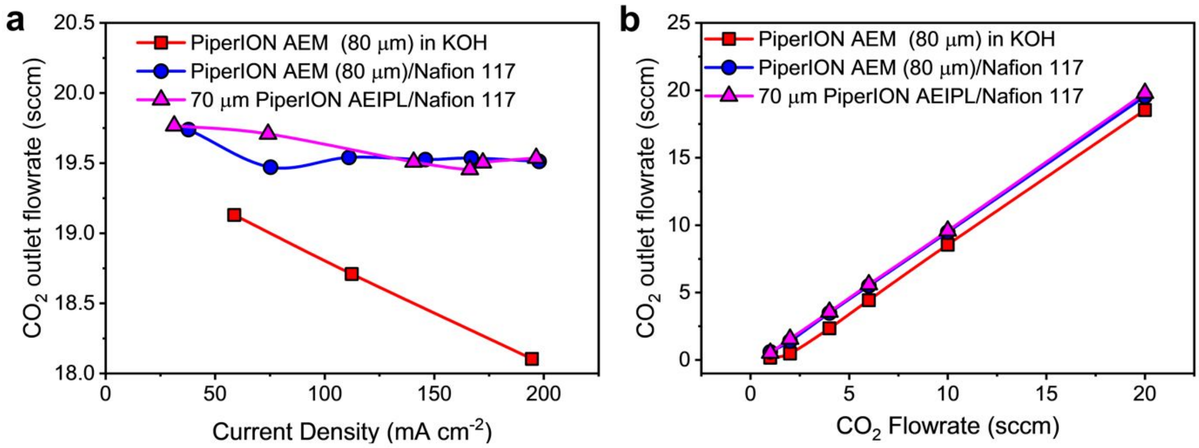

Figure S4. Comparison of CO_2 outlet flow rate from cathodic chamber for electrolyzer configurations; (a) CO_2 outlet flow rate vs. current density at 20 SCCM feed flow rate, and (b) CO_2 outlet flow rate vs. feed flow rate at current density of 150 mA cm^{-2}.

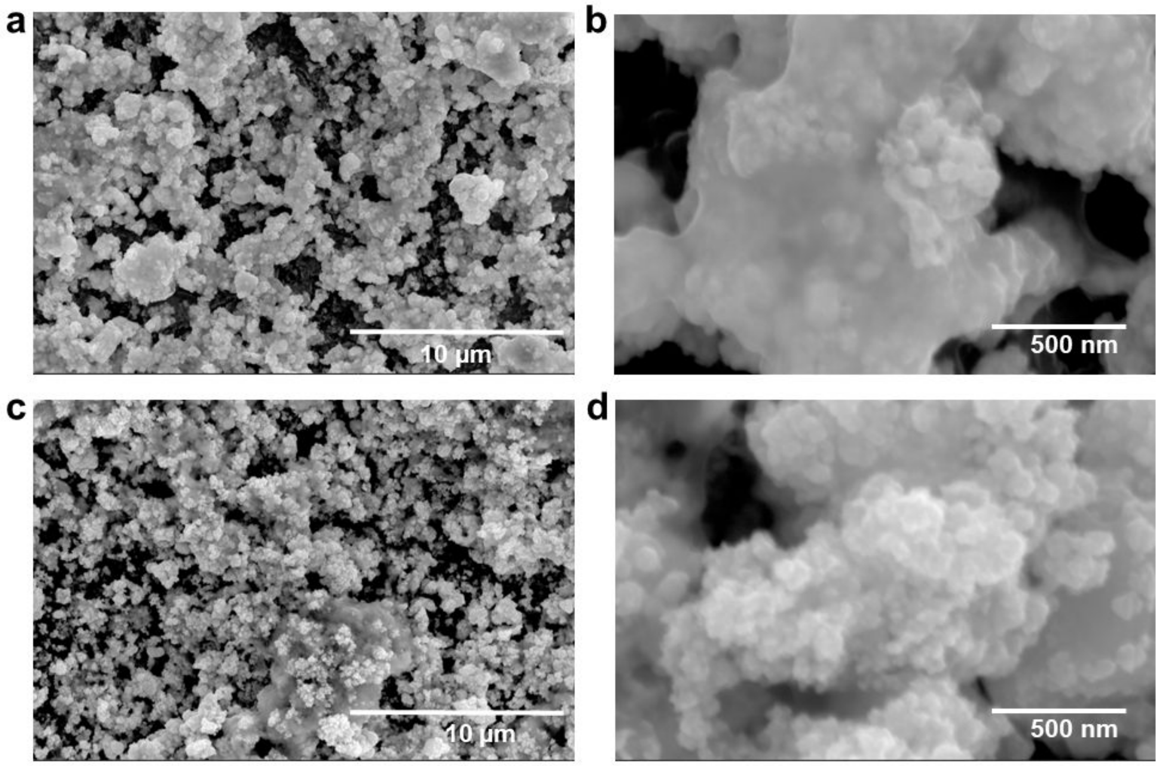

Figure S5. SEM images of copper NPs-coated GDE (a and b) before electrochemical CO_2 reduction reaction and (c and d) after 100 h of electrochemical CO_2 reduction reaction in AEIPL BPMEA electrolyzer.

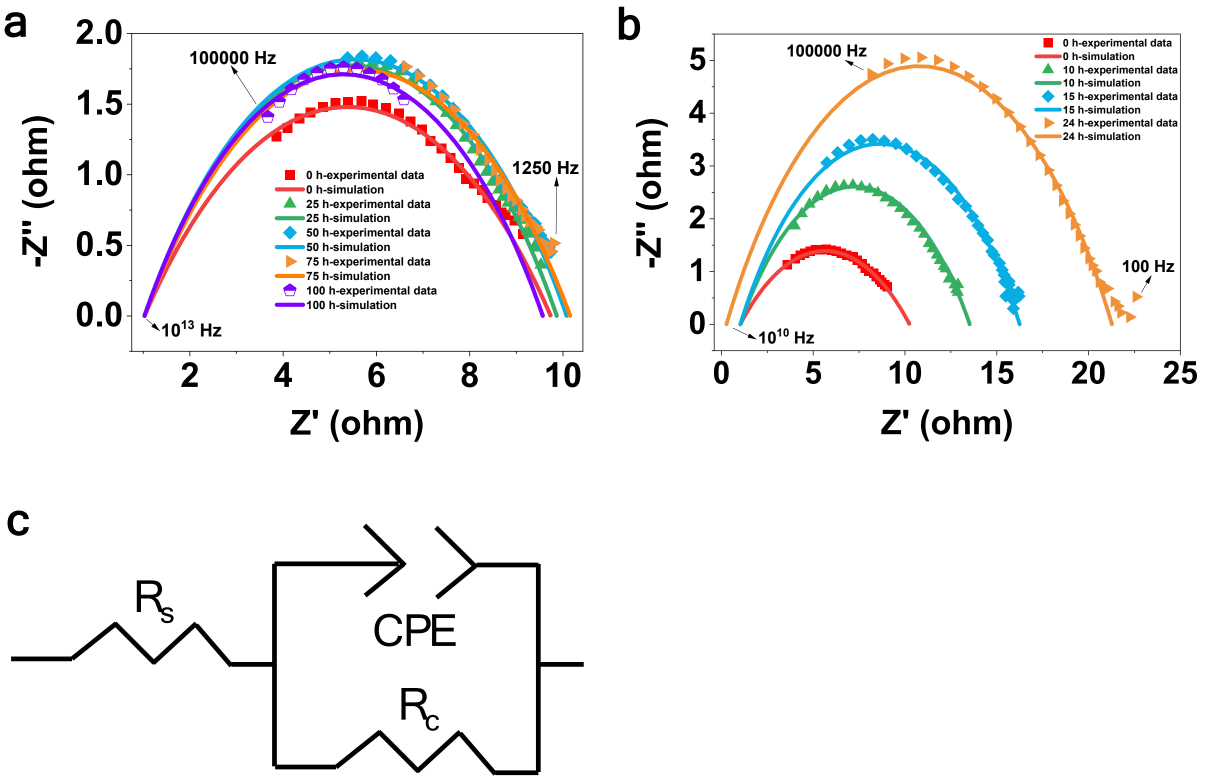

Figure S6. Nyquist plots of a) PiperION AEIPL/Nafion 117 and b) PiperION AEM/Nafion 117 BPM measured during long-term stability, and c) the modified Randles circuit used for data fitting and simulation.

Table S1: Comparison of present work with different CO_2 crossover inhibition methods.[1-6]

S. No	Major CO_2R Product FE	Crossover (sccm)	Cell voltage (V)	Current Density (mA cm^{-2})	Stability (h)	Method for CO_2 crossover inhibition	Challenge	Ref.
1	40% C_2H_4	0.2	4.0	100	8	Ionomer coating on rough catalyst-loaded GDE	Ionomer coating layer is prone to cracks while preparation	1
2	51% C_2H_4	0.03	3.8	100	200	Micro-channeled solid electrolyte	Complex fabrication procedure	2
3	80% CO	NA	2.9	100	NA	Acidic integrated channel layer interface between the AEL and CEL	Design lacks microporosity, which may lead to delamination	3
4	42% C_2H_4	0.1	3.8	200	50	BPM electrolyzer with a stationary buffer catholyte layer (65 μm thick 0.5 M K_2SO_4 layer)	The cell's stability was limited may be due to delamination at the BPM interface	4
5	90% CO	0.2	3.8	100	750	Porous solid electrolyte between AEM and CEM	Introduces an extra resistance and energy penalty to the electrolyzer operation	5
6	50 % C_2H_4	NA	4.2	300	1000	BPMEA system under an elevated temperature (e.g., 60 °C)	Elevated temperatures promote the thermal degradation of AEM, ultimately leading to the long-term instability of the electrolyzer	6
7	42% C_2H_4 (Stable at 17%)	0.2	4.5	200	100	Ionomer coating on smooth Nafion membrane	Simple and highly reproducible method	Present work

Table S2: Properties and cation structures of commercial anion exchange membranes.[7-9]

Membrane Trademark	OH^- ion conductivity (mS/cm)	Bader charge on the N atom (e)	IEC (meq/g)	Cation	Thickness (μm)	Cation Structure	Backbone structure
AEM#1	80	0.15 - 0.25	2.3-2.6	Benzimidazolium	50	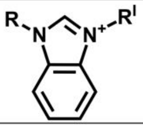	Polyaromatic (Polyphenylene oxide)
AEM#2	40	0.60 - 0.80	1.6-2.1	Quaternary ammonium	50	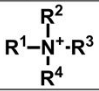	Aromatic polysulphone derivatives
AEM#3	80	0.10 - 0.20	1.1	Imidazolium	50	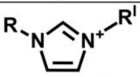	Flouropolymer (PTFE)
AEM#4 (PiperION)	65-90	0.60 - 0.80	2.0-2.3	Piperidinium	80	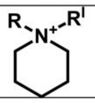	Polyaromatic (Polyphenylene)

Table S3: R_{ct} and R_s values of PiperION AEIPL/Nafion 117

S. No	Time (h)	R_{ct} (Ω)	R_s (Ω)
1	0	8.714	1.029
2	25	8.824	1.028
3	50	9.053	1.028
4	75	9.128	1.028
5	100	8.546	1.028

Table S4: R_{ct} and R_s values of PiperION AEM/Nafion 117 BPM

S. No	Time (h)	R_{ct} (Ω)	R_s (Ω)
1	0	3.528	1.026
2	10	12.564	1.028
3	15	15.227	1.025
4	24	21.042	0.272

References

(1) O'Brien, C. P.; Miao, R. K.; Liu, S.; Xu, Y.; Lee, G.; Robb, A.; Huang, J. E.; Xie, K.; Bertens, K.; Gabardo, C. M.; et al. Single Pass CO_2 Conversion Exceeding 85% in the Electrosynthesis of Multicarbon Products via Local CO_2 Regeneration. *ACS Energy Letters* **2021**, *6* (8), 2952-2959. DOI: 10.1021/acsenergylett.1c01122.

(2) Xu, Y.; Miao, R. K.; Edwards, J. P.; Liu, S.; O'Brien, C. P.; Gabardo, C. M.; Fan, M.; Huang, J. E.; Robb, A.; Sargent, E. H.; et al. A microchanneled solid electrolyte for carbon-efficient CO_2 electrolysis. *Joule* **2022**, *6* (6), 1333-1343. DOI: https://doi.org/10.1016/j.joule.2022.04.023.

(3) Brückner, S.; Ju, W.; Strasser, P. Efficient Forward-Bias Bipolar Membrane CO_2 Electrolysis in Absence of Metal Cations. *Advanced Energy Materials 15* (25), 2500186. DOI: https://doi.org/10.1002/aenm.202500186.

(4) Xie, K.; Miao, R. K.; Ozden, A.; Liu, S.; Chen, Z.; Dinh, C.-T.; Huang, J. E.; Xu, Q.; Gabardo, C. M.; Lee, G.; et al. Bipolar membrane electrolyzers enable high single-pass CO_2 electroreduction to multicarbon products. *Nature Communications* **2022**, *13* (1), 3609. DOI: 10.1038/s41467-022-31295-3.

(5) Kim, J. Y. T.; Zhu, P.; Chen, F.-Y.; Wu, Z.-Y.; Cullen, D. A.; Wang, H. Recovering carbon losses in CO_2 electrolysis using a solid electrolyte reactor. *Nature Catalysis* **2022**, *5* (4), 288-299. DOI: 10.1038/s41929-022-00763-w.

(6) She, X.; Zhai, L.; Wang, Y.; Xiong, P.; Li, M. M.-J.; Wu, T.-S.; Wong, M. C.; Guo, X.; Xu, Z.; Li, H.; et al. Pure-water-fed, electrocatalytic CO_2 reduction to ethylene beyond 1,000 h stability at 10 A. *Nature Energy* **2024**, *9* (1), 81-91. DOI: 10.1038/s41560-023-01415-4.

(7) Henkensmeier, D.; Najibah, M.; Harms, C.; Žitka, J.; Hnát, J.; Bouzek, K. Overview: State-of-the Art Commercial Membranes for Anion Exchange Membrane Water Electrolysis. *Journal of Electrochemical Energy Conversion and Storage* **2020**, *18* (2). DOI: 10.1115/1.4047963 (acccessed 6/11/2025).

(8) Schmidt, J.; Krekeler, C.; Dommert, F.; Zhao, Y.; Berger, R.; Site, L. D.; Holm, C. Ionic Charge Reduction and Atomic Partial Charges from First-Principles Calculations of 1,3-Dimethylimidazolium Chloride. *The Journal of Physical Chemistry B* **2010**, *114* (18), 6150-6155. DOI: 10.1021/jp910771q.

(9) OpenAI. Response to "Bader charge on N atom of 1,3 dimethylimidazolium cation, tetramethylammonium cation, 1,1 dimethyl piperidium cation, and 1,3 dimethybenzimidazolium cation," https://chat.openai.com/. **7 March 2026**.
